# Image quality evaluation of dual-layer spectral CT in comparison to single-layer CT in a reduced-dose setting

**DOI:** 10.1007/s00330-020-06894-7

**Published:** 2020-05-11

**Authors:** Thuy Duong Do, Stephan Rheinheimer, Hans-Ulrich Kauczor, Wolfram Stiller, Tim Weber, Stephan Skornitzke

**Affiliations:** 1 Clinic for Diagnostic and Interventional Radiology (DIR), Heidelberg University Hospital, Im Neuenheimer Feld 110, 69120 Heidelberg, Germany; 2 Translational Lung Research Center (TLRC), Member of the German Center for Lung Research (DZL), Heidelberg, Germany

**Keywords:** Tomography, X-ray computed, Radiation exposure, Thorax, Abdomen

## Abstract

**Objectives:**

To quantitatively and qualitatively evaluate image quality in dual-layer CT (DLCT) compared to single-layer CT (SLCT) in the thorax, abdomen, and pelvis in a reduced-dose setting.

**Methods:**

Intraindividual, retrospective comparisons were performed in 25 patients who received at least one acquisition of all three acquisition protocols SLCT_low_ (100 kVp), DLCT_high_ (120 kVp), and DLCT_low_ (120 kVp), all covering the venous-phase thorax, abdomen, and pelvis with matched CTDI_vol_ between SLCT_low_ and DLCT_low_. Reconstruction parameters were identical between all scans. Image quality was assessed quantitatively at 10 measurement locations in the thorax, abdomen, and pelvis by two independent observers, and subjectively with an intraindividual forced choice test between the three acquisitions. Dose-length product (DLP) and CTDI_vol_ were extracted for dose comparison.

**Results:**

Despite matched CTDI_vol_ in acquisition protocols, CTDI_vol_ and DLP were lower for SLCT_low_ compared to DLCT_low_ and DLCT_high_ (DLP 408.58, 444.68, 647.08 mGy·cm, respectively; *p* < 0.0004), as automated tube current modulation for DLCT_low_ reached the lower limit in the thorax (mean 66.1 mAs vs limit 65 mAs). Noise and CNR were comparable between SLCT_low_ and DLCT_low_ (*p* values, 0.29–0.51 and 0.05–0.20), but CT numbers were significantly higher for organs and vessels in the upper abdomen for SLCT_low_ compared to DLCT_low_. DLCT_high_ had significantly better image quality (Noise and CNR). Subjective image quality was superior for DLCT_high_, but no difference was found between SLCT_low_ and DLCT_low_.

**Conclusions:**

DLCT_low_ showed comparable image quality to SLCT_low_, with the additional possibility of spectral post-processing. Further dose reduction seems possible by decreasing the lower limit of the tube current for the thorax.

**Key Points:**

• *Clinical use of reduced-dose DLCT is feasible despite the required higher tube potential.*

• *DLCT with reduced dose shows comparable objective and subjective image quality to reduced-dose SLCT.*

• *Further dose reduction in the thorax might be possible by adjusting mAs thresholds.*

## Introduction

The introduction of dual-layer detector technology in computed tomography (CT) enabled the acquisition of spectral data for all performed scans without the need of an additional CT x-ray tube or additional acquisitions. Dual-layer spectral CT (DLCT) acquisitions allow material decomposition (virtual non-contrast, iodine-only imaging, and effective atomic numbers) as well as the calculation of virtual monoenergetic images. Several clinical studies have already been performed showing the advantages of DLCT for head CT for imaging intracerebral lesions and hemorrhage, for thoracic CT, for vertebral CT for differentiating bone lesions, and for abdominal CT angiographies for improved delineation of visceral arteries [1–5]. However, for the image acquisition of such data, a tube potential of either 140 kVp or 120 kVp is necessary to allow for spectral decomposition under the exploitation of the energy-specific x-ray absorption of different materials. In contrast to changes in tube current, changes in tube potential have a non-linear effect on radiation dose: in comparison to 80 kVp, the x-ray tube output (i.e., air kerma or exposure) is 1.5 times higher for 100 kVp, 2.5 times higher for 120 kVp, and 3.4 times higher for 140 kVp [6].

While patient radiation exposure has long been a topic of interest in CT, as CT accounts for 49–66% of overall patient radiation exposure, this interest has recently led to new regulations in the European Union via the EURATOM directive, with the deadline for implementation into federal law in 2018 [7, 8]. Based on surveys, diagnostic reference levels (DRLs) at the 75th percentile of dose distribution have been defined as the limits of appropriate practice for CT acquisitions. In our institution, acquisition protocols for the thorax, abdomen, and pelvis have been previously optimized to reduce patient radiation exposure beyond these legal requirements. This was achieved, in part, by reducing tube potential from 120 to 100 kVp for single-layer CT (SLCT), resulting in an average patient dose 60% below the federal DRL for the thorax, abdomen, and pelvis, which is a DLP of 1000 mGy·cm and a CTDI_vol_ of 13 mGy.

With the installation of a novel DLCT, vendor’s recommended settings were applied including a tube potential of 120 kVp (acquisition protocol DLCT_high_), which led to an increase in radiation exposure in comparison to previous reduced-dose SLCT protocols (acquisition protocol SLCT_low_), albeit still remaining below the applicable diagnostic reference levels. After initial experience with DLCT_high_, the tube current was reduced (acquisition protocol DLCT_low_) to match the CTDI_vol_ of the reduced-dose protocol previously performed on the SLCT scanner at 100 kVp (SLCT_low_).

The aim of the study was to quantitatively and qualitatively compare the image quality and radiation exposure of the SLCT acquisition protocol with reduced dose, SLCT_low_ (41% of the national DRL), and the DLCT acquisition protocol with reduced dose, DLCT_low_ (44% of the DRL). Thus, by comparing DLCT_low_ and SLCT_low_, we want to aid in the deployment of protocols with reduced dose in DLCT with image quality comparable to previous low-tube potential SLCT_low_, while allowing to benefit from the spectral information in a clinical setting.

## Material and methods

### Ethics approval and consent

This retrospective study was approved by the institutional review board. The need for written informed consent was waived.

### Patient selection

**Fig. 1:**
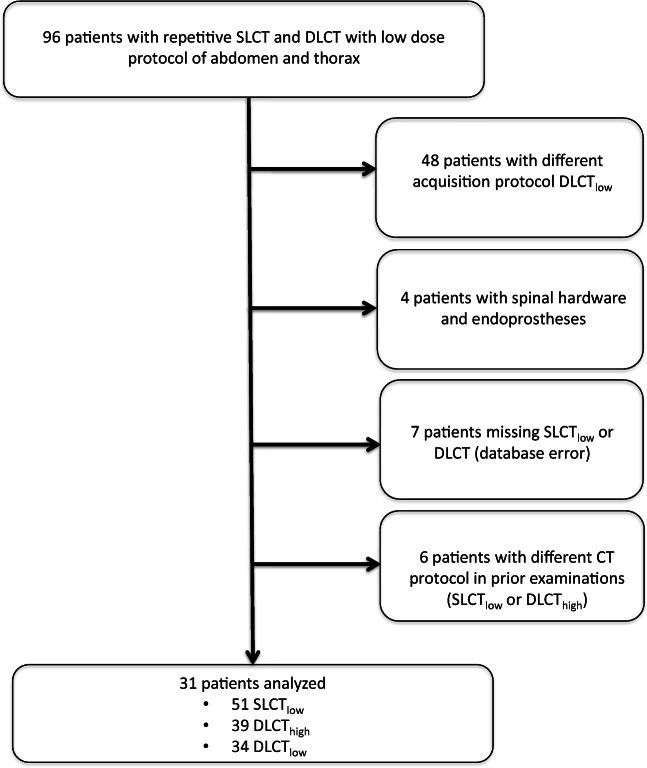
Flowchart illustrating study inclusion and exclusion criteria. In total, 31 patients could be included into the study with repeated CT scans consisting of 51 SLCT_low_, 39 DLCT_high_, and 34 DLCT_low_

Ninety-six patients who underwent low-dose DLCT (DLCT_low_) and previous low-dose SLCT_low_ with clinical indication for oncological staging, since October 2018, were screened for inclusion into the study (Fig. 1). Intensive care patients and obese patients were excluded from the study, as low-dose protocols are not routinely used in these circumstances and obese patients require adapted acquisition protocols to guarantee adequate image quality. Arterial-phase acquisitions or venous-phase examinations with additional arterial angiography phase were not considered for the study to ensure comparability of the evaluated acquisitions, as those are to prone to effects of contrast agent volume, injection speed of the contrast agent, and circulation and cardiac output. In consequence, only patients with a routine CT acquisition of the thorax, abdomen, and pelvis in the venous phase were included into the study. All patients received a similar total amount of contrast agent in all their examinations with a maximum intraindividual difference of 5 ml. Patients with diffuse liver parenchyma diseases (liver cirrhosis and liver metastases), liver perfusion deficit, or intracorporal metal implants (e.g., hip prosthesis or spinal hardware) were excluded from the study. Furthermore, non-standard CT examinations as defined by differences in the acquisition protocol were excluded from the study. In summary, 31 patients were included into the study, who previously underwent 51 SLCT_low_ examinations, 39 DLCT_high_ examinations, and 34 DLCT_low_ examinations with adjusted parameters (Fig. 1). The mean interval between the acquisitions with the different acquisition protocols was 224.6 ± 89.8 days (median, 190 days) between SLCT_low_ and DLCT_high_ and 182.2 ± 70.5 days (median, 183.5 days) between DLCT_high_ and DLCT_low_. A subgroup of 25 patients had been examined with all three CT acquisition protocols and was used for qualitative analysis.

### CT acquisition parameters

CT acquisitions were performed with a standard acquisition protocol for oncological staging covering the thorax, abdomen, and pelvis by a single continuous acquisition (all three scan protocols). Acquisition parameters of SLCT_low_ (iCT, Philips Healthcare), DLCT_low_, and DLCT_high_ (IQon Spectral CT, Philips Healthcare) are shown in Table 1. SLCT_low_ was performed with a tube potential of 100 kVp, while all DLCTs were performed with a tube potential 120 kVp. For DLCT_high_, standard acquisition settings were used as per the manufacturer’s presets. For DLCT_low_, the automated tube current modulation (DoseRight 3D-DOM, Philips Healthcare) was adjusted by reducing dose right index (DRI) from 17 to 13 to match the SLCT_low_ protocol, achieving a similar predicted CTDI_vol_, as displayed when editing the acquisition protocol in the CT scanner interface (DLCT_low_ 7.5 mGy, SLCT_low_ 7.8 mGy). Accordingly, predicted average mAs was lower for DLCTl_ow_ (74 mAs) compared to DLCT_high_ (116 mAs).

**Table 1:** CT acquisition parameters for SLCT_low_, DLCT_low_, and DLCT_high_

	SLCT_low_	DLCT_low_	DLCT_high_
Tube potential	100 kVp	120 kVp	120 kVp
Automated tube current modulation (dose right index)	14	13	17
Predicted CTDI_vol_	7.8 mGy	7.5 mGy	11.5 mGy
Predicted average mAs	174 mAs	74 mAs	116 mAs
Minimum mAs	65 mAs	65 mAs	65 mAs
Maximum mAs	300 mAs	300 mAs	350 mAs

Collimation (single collimation width 0.625 mm and total collimation width 40 mm) and pitch (0.798) was identical for all scans.

All examinations were conducted in craniocaudal direction and supine position, with automatic exposure control as described above. For all acquisitions, iohexol contrast agent (AccupaqueTM 350, GE Healthcare) was used. Contrast agent application was performed using a power injector with an injection rate of 3 ml/s. A routine biphasic contrast-injection scan protocol was used covering the thorax, abdomen, and pelvis in the venous phase. The first contrast agent injection and saline solution chaser bolus was followed by a 30-s break and a second contrast agent injection with a saline solution chaser bolus. Then the CT acquisition was performed 60 s after the second contrast agent injection. The combined acquisition of thorax and abdominal region in the venous phase achieves better delineation of the lymph nodes, thoracic wall, and mediastinal soft tissue with less artifacts than a thoracic arterial phase for oncological staging [9]. As previously stated, only patients who received a venous-phase CT acquisition of the thorax, abdomen, and pelvis were included in the study.

### Image reconstruction and post-processing

For all examinations, axial series with a slice thickness of 3 mm and increment of 1.5 mm were reconstructed. Images were reconstructed with a vendor-specific iterative reconstruction algorithm, IMR at level 1 (Iterative Model Reconstruction, Philips Healthcare), soft-tissue setting, and a standard abdomen setting (window center/width 40/400 HU).

### Dose and image analysis

Quantitative and qualitative analysis were performed by two radiologists with 7 and 6 years of experience in abdominal radiology. For quantitative analysis, all acquisitions were included. For qualitative analysis, the most recent examination of each acquisition protocol (SLCT_low_, DLCT_high_, and DLCT_low_) was included and only patients that were scanned with all three acquisition protocols were included.

**Fig. 2:**
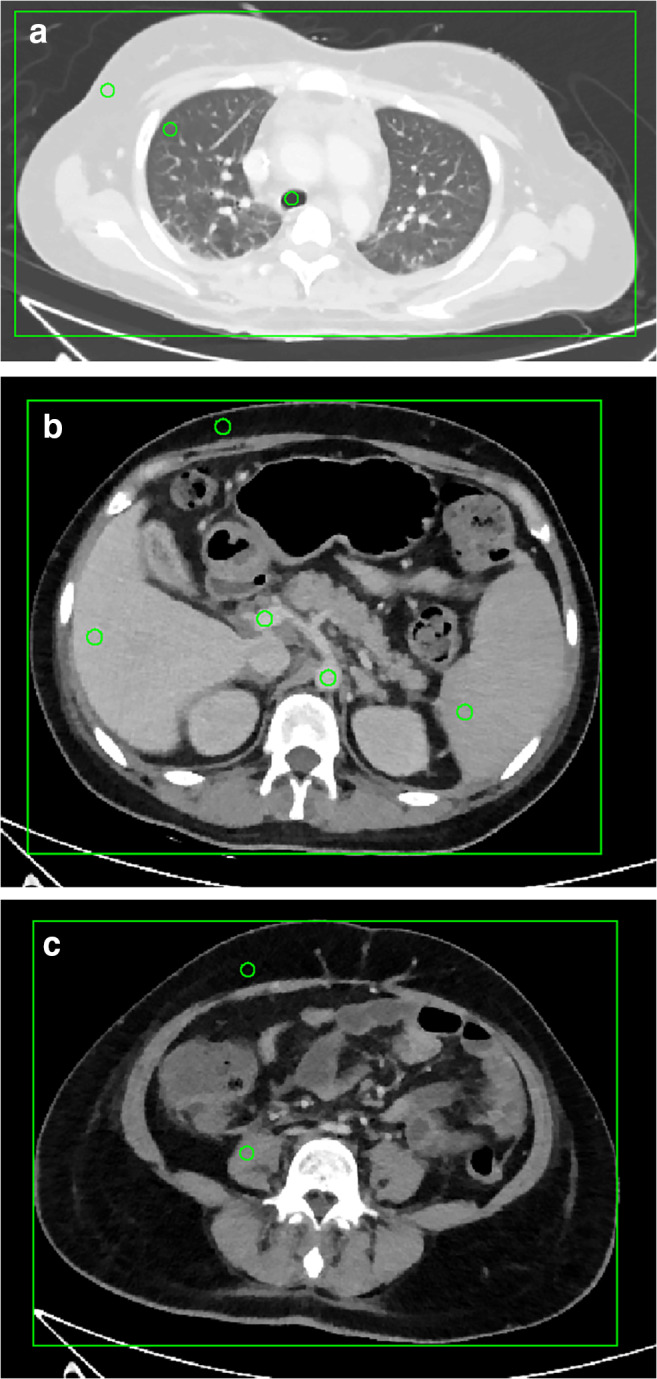
Image examples of regions of interest (ROIs) used for the quantitative evaluation in the thorax at the height of the tracheal bifurcation (**a**), the upper abdomen at the height of the portal vein (**b**), and the lower abdomen at the level of the lumbar spine L4 (**c**). Rectangle for measurements of the lateral and anterior-posterior diameter of the torso

Patients’ anterior-posterior and lateral diameters were measured separately for the thorax at the level of tracheal bifurcation, the upper abdomen at the level of the portal vein, and the lower abdomen at the level of lumbar spine L4 (Fig. 2). Total scan lengths were calculated from the images.

Quantitative image analysis was performed by placement of regions of interest (ROIs) in axial slices in the following positions (Fig. 2):

- Level of the tracheal bifurcation: in the subcutaneous fat, peripheral lung parenchyma, and air within the trachea
- Level of the portal vein: in the subcutaneous fat of the right upper abdomen, liver parenchyma, portal vein, aorta, and spleen
- Level of the lumbar spine L4: in the subcutaneous fat of the right lower abdomen and psoas muscle

For all three axial slices, the slice-specific tube current-exposure time product, lateral, and anterior-posterior torso diameter were determined. An in-house developed software was used for semiautomatic quantitative image quality analysis to maintain constant ROI area size of 50 mm^2^ in order to enhance reproducibility and time-efficient evaluation [10]. Noise was defined as the standard deviation (SD) of CT numbers of the subcutaneous fat on the right side of the ventral thorax or abdomen for improved comparability. Mean and SD of CT numbers were recorded and CNR was calculated as follows:

$$ \left(\mathrm{I}\right)\ \mathrm{CNR}=\left({\mathrm{Mean}}_{\mathrm{ORGAN}}-{\mathrm{Mean}}_{\mathrm{FAT}}\right)/{\mathrm{SD}}_{\mathrm{FAT}} $$

The following ROIs were used to calculate CNR in comparison to the subcutaneous fat on their respective axial slice: lung parenchyma (thorax), liver parenchyma (upper abdomen), and psoas muscle (lower abdomen).

**Fig. 3:**
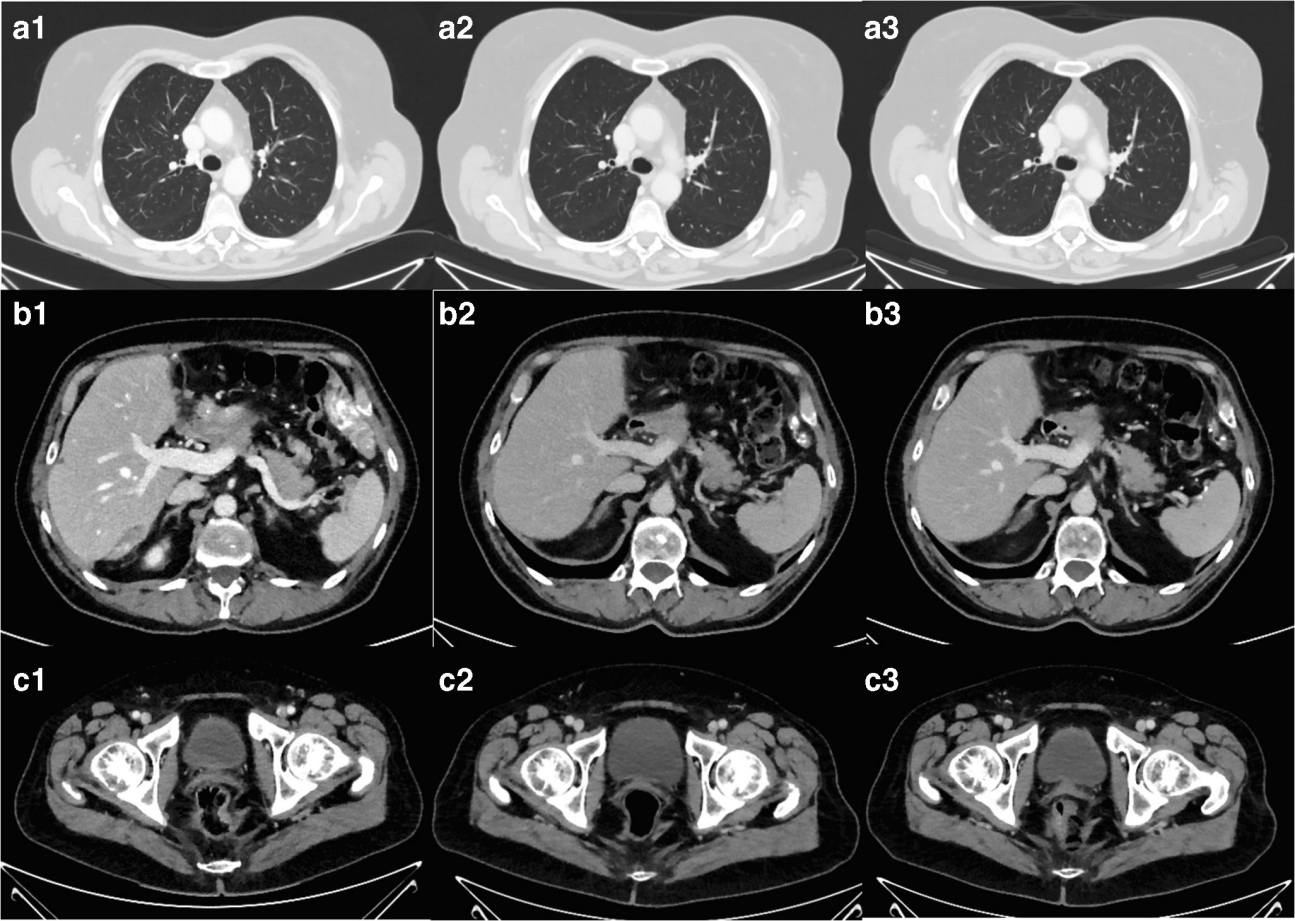
Image examples from the same patient from conventional reduced-dose SLCT_low_ (column 1), DLCT_low_ (column 2), and DLCT_high_ (column 3) from thorax (**a**), upper abdomen (**b**), and pelvis (**c**). DLCT_high_ (column 2) showed best quantitative and qualitative analysis whereas SLCT_low_ (column 1) and DLCT_low_ (column 3) showed comparable results

For qualitative image evaluation comparing SLCT_low_, DLCT_high_, and DLCT_low_ (Fig. 3), a forced choice method was used for a blinded and randomized side-by-side review. For randomization, both the order of patients and evaluated series were randomized by the statistician, who was not involved in qualitative image evaluation, using the random function of EXCEL (Microsoft). Two observers independently performed the qualitative analysis on conventional CT images reconstructed from all three acquisition protocols. First, images from all three acquisition protocols were separately ranked for three body regions covered by the acquisition protocols taking into account the following criteria [11]:

- Thorax: noise in the subcutaneous fat of the right thorax, delineation of trachea, bronchi, and spinal cord
- Upper abdomen: noise in the right-sided subcutaneous fat, delineation of portal vein, aorta, and spinal cord
- Lower abdomen: noise in the right-sided subcutaneous fat, delineation of gluteal muscles, and spinal cord

Based on the rankings for the three body regions, unique ranks from 1 to 3 were allocated to the evaluated image series (i.e., observes were forced to choose which image has the best image quality compared to the others) as a total score, whereby a rank of 1 corresponded to the best image quality.

DLPs, CTDI_vol_, and overall tube current-time product were extracted from the patient’s dose report images sent to the picture archiving system. Size-specific dose estimates (SSDEs) for each patient and each body region were calculated according to the recommendations of the American Association of Physicsts in Medicine Task Group 204 [12]. CTDI_vol_ and DLP are frequently used parameters for estimation of a patient’s radiation exposure. However, SSDE is a more precise method for the patient’s absorbed dose as it takes the patient’s size into account [13]. To calculate SSDE for the individual anatomical region, a region-specific CTDI_vol,region_ was estimated by dividing the CTDI_vol_ for the complete acquisition by the average tube current-time product and multiplying it with the tube current-time product of the representative axial slice of each region (Fig. 2):

$$ \left(\mathrm{II}\right)\ {\mathrm{CTDI}}_{\mathrm{vol},\mathrm{region}}={\mathrm{CTDI}}_{\mathrm{vol}}\cdot \mathrm{average}\ {\mathrm{mAs}}_{\mathrm{total}}/\mathrm{average}\ {\mathrm{mAs}}_{\mathrm{region}} $$

The resulting estimated region-specific CTDI_vol,region_ was multiplied with the recommended conversion coefficient depending on the lateral and AP diameter of the selected anatomical region [13].

### Statistical analysis

Statistical analysis was performed using SAS Version 9.4 (SAS Institute Inc.) and SPSS Version 19.0 (IBM). Descriptive statistics were calculated, determining means and standard deviations for normal distributed data. CT numbers, image noise approximated by the standard deviation of measured CT numbers, and CNR were analyzed for differences depending on the CT scanner and the acquisition protocol using a mixed model for unbalanced analysis of variances for repeated measures.

Qualitative ratings of image quality were analyzed for differences depending on the CT scanner and the acquisition protocol using non-parametric tests (Friedman test stratified for patients and Wilcoxon signed-rank test in a multistage design).

For the evaluation of the inter-rater agreement of the quantitative analysis, the intraclass correlation coefficient (ICC) was used and for the qualitative analysis Cohen’s kappa was calculated and classified according to Landis and Koch [14]. The significance level for statistical testing was set at *p* < 0.05.

## Results

### Quantitative analysis

Although the average scan length of DLCT_low_ was significantly shorter than that for SLCT_low_ (*p* > 0.0001) with an average difference of 13.8 mm, DLP for DLCT_low_ was significantly higher due to higher CTDI_vol_ of DLCT_low_ in comparison to SLCT_low_ (444.68 mGy·cm and 408.58 mGy·cm, respectively; *p* = 0.0004) (Table 2). CTDI_vol_ of DLCT_low_ (6.57 mGy) was 30% lower than that of DLCT_high_ (9.34 mGy; *p* < 0.0001). Despite the efforts to match radiation dose, average CTDI_vol_ was 14.4% higher for DLCT_low_ compared to SLCT_low_ (*p* < 0.0001). Similar to CTDI_vol_ and DLP, the SSDE was lowest for SLCT_low_ while SSDE of DLCT_low_ was significantly less than DLCT_high_ for all body regions (*p* < 0.0001). DLCT_high_ had significantly higher CTDI_vol_ (*p* < 0.0001) and higher DLP (*p* < 0.0001) resulting from higher tube potential in comparison to SLCT_low_, despite comparable scan length to SLCT_low_ (*p* = 0.56).

**Table 2:** Evaluation of patient radiation exposure: means of scan length, CTDI_vol_, DLP, and SSDE of SLCT_low_, DLCT_high_, and DLCT_low_

	Scan length (mm)	CTDI_vol_ (mGy)	DLP (mGy·cm)	SSDE Thorax (mGy)	SSDE upper abdomen (mGy)	SSDE lower abdomen (mGy)
SLCT_low_	640.71 ± 39.52	5.74 ± 1.26	408.58 ± 107.58	5.00 ± 1.20	8.41 ± 1.79	7.28 ± 1.72
DLCT_low_	626.91 ± 38.77	6.57 ± 0.72	444.68 ± 57.63	7.28 ± 0.66	9.19 ± 1.04	8.30 ± 0.66
DLCT_high_	642.04 ± 37.18	9.34 ± 2.13	647.08 ± 155.12	7.92 ± 0.91	14.11 ± 2.62	11.52 ± 1.96

A decrease of the mean lateral diameter of patients over time was observed, with a maximum difference/change of 10.3 mm (Table 3). There was also a statistically significant difference of both the lateral thoracic and lower abdominal diameter between the first (and earliest) examination with SLCT_low_ in comparison to the acquisitions with DLCT_high_ (*p* < 0.0006 and 0.0029, respectively), but the mean difference was only 5.6 mm (thorax) and 9.2 mm (lower abdomen). No significant differences in anterior-posterior (AP) diameter were observed for all three abdominal regions.

**Table 3:** Evaluation of automatic exposure control: means and standard deviations of patients’ lateral and anterior-posterior (AP) diameter, applied tube current-time product at the measurement location, and results of statistical testing. Statistical significance of results is indicated by italics

Anatomic location	SLCT_low_ (*)	DLCT_low_ (**)	DLCT_high_ (***)	*p* value		
				* vs. **	* vs. ***	** vs. ***
Thorax						
Lateral (mm)	389.9 ± 31.3	379.6 ± 31.7	384.3 ± 31.6	*0.0054*	*0.0006*	0.53
AP (mm)	231.3 ± 31.3	229.6 ± 28.5	237.5 ± 31.0	0.70	0.74	0.51
mAs	98.8 ± 29.1	66.1 ± 3.0	74.3 ± 12.6	*< 0.0001*	*< 0.0001*	0.11
Upper abdomen						
Lateral (mm)	326.5 ± 23.6	320.4 ± 25.9	326.3 ± 27.1	*0.027*	0.86	0.052
AP (mm)	247.2 ± 29.8	242.1 ± 32.0	252.7 ± 35.9	0.56	0.20	0.087
mAs	151.9 ± 41.5	77.4 ± 14.4	123.9 ± 35.4	*< 0.0001*	*< 0.0001*	*< 0.0001*
Lower abdomen						
Lateral (mm)	348.5 ± 36.9	339.3 ± 36.0	342.7 ± 39.5	*0.0055*	*0.0029*	0.88
AP (mm)	236.6 ± 31.3	233.7 ± 34.4	240.9 ± 35.4	0.68	0.46	0.77
mAs	174.1 ± 58.5	80.7 ± 25.4	116.9 ± 34.4	*< 0.001*	*< 0.0001*	*< 0.0001*

**Fig. 4:**
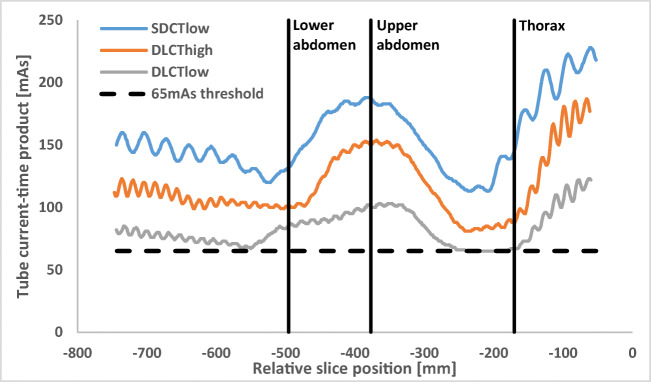
Example of tube current-time product curves for the three evaluated acquisition protocols in one patient. Note the changes in tube current in the different anatomical regions caused by the automated exposure control, as well as the changes during the rotation of the x-ray tube around the patient table

In general, the lowest tube current-time product was found for DLCT_low_ in all three regions, showing a statistically significant difference compared to DLCT_high_ in the lower and upper abdomen, but not in the thorax (Table 3). It has to be noted that for the thorax, the average tube current-time product of DLCT_low_ was 66.1 mAs, which is close to the minimum mAs of the tube current modulation preset at 65 mAs (Fig. 4). Also, mAs for the upper and lower abdomen was lowest for DLCT_low_, at approximately 49–51% of SLCT_low_.

There was no significant difference in noise between SLCT_low_, DLCT_low_, and DLCT_high_ for the thorax and upper abdomen (*p* between 0.08 and 0.61). No significant difference in noise was found between SLCT_low_ and DLCT_low_ in the lower abdomen (*p* = 0.51). In general, noise was lowest for DLCT_high_, but the difference was only statistically significant in the lower abdomen (*p* < 0.05) (Table 4). No significant difference was found for CNR in the thorax and upper abdomen between SLCT_low_ and DLCT_low_. In the lower abdomen, CNR for DLCT_low_ was significantly lower than that for DLCT_high_, but no significant difference was found between DLCT_low_ and SLCT_low_. As for CT numbers, highest values were observed for vessels, e.g. aorta and portal vein, in comparison to organ parenchyma due to the use of contrast agent (Table 5). CT numbers of vessels in SLCT_low_ were significantly higher than those for both DLCT_low_ and DLCT_high_ which can be attributed to higher CT numbers observed for contrast agents at the lower tube potential of 100 kVp versus 120 kVp. CT numbers for DLCT_high_ and DLCT_low_ in organ parenchyma did not show significant differences, except for the liver, which can show considerable contrast agent uptake, where the mean difference was 13.7 HU between SLCT_low_ and DLCT_high_.

**Table 4:** Results of the quantitative image quality evaluation: image noise and CNR. Statistical significance of results is indicated by italics

Quantitative parameters	SLCT_low_ (*)	DLCT_low_ (**)	DLCT_high_ (***)	*p* value		
Anatomic location				* vs. **	* vs. ***	** vs. ***
Thorax						
Noise (fat) (HU)	5.4 ± 2.0	5.2 ± 2.6	5.0 ± 2.2	0.29	0.08	0.53
CNR (lung)	− 157.8 ± 52.7	− 165.4 ± 45.6	− 173.9 ± 51.5	0.20	*0.020*	0.33
Upper abdomen						
Noise (fat) (HU)	5.9 ± 1.6	6.1 ± 2.2	5.6 ± 1.6	0.32	0.61	0.16
CNR (liver)	41.7 ± 12.7	38.6 ± 12.3	39.8 ± 11.8	0.05	0.10	0.71
Lower abdomen						
Noise (fat) (HU)	5.1 ± 1.5	5.1 ± 1.2	4.6 ± 1.1	0.51	*0.0018*	*0.02*
CNR (psoas)	37.3 ± 10.8	35.0 ± 8.6	39.5 ± 10.4	0.18	0.12	*0.0085*

**Table 5:** Results of the quantitative image quality evaluation: CT numbers of evaluated organs and vessels in the thorax, upper abdomen, and lower abdomen. Statistical significance of results is indicated by italics

Quantitative parameters	SLCT_low_ (*)	DLCT_low_ (**)	DLCT_high_ (***)	*p* value		
Anatomic location				* vs. **	* vs. ***	** vs. ***
Thorax						
Subcutaneous fat (HU)	− 115.7 ± 12.0	− 110.5 ± 12.9	− 113.0 ± 12.1	*0.0013*	*0.035*	0.23
Lung (HU)	− 875.5 ± 39.8	− 877.1 ± 43.6	− 877.1 ± 49.2	0.54	0.80	0.42
Trachea (HU)	− 987.8 ± 14.0	− 991.9 ± 7.2	− 993.9 ± 7.6	*0.0018*	*0.0002*	0.58
Upper abdomen						
Subcutaneous fat (HU)	− 114.4 ± 10.4	− 104.7 ± 14.3	− 106.6 ± 16.2	*< 0.0001*	*< 0.0001*	0.81
Liver (HU)	113.0 ± 18.7	106.7 ± 18.8	99.3 ± 22.3	*< 0.0001*	*< 0.0001*	*0.0015*
Portal vein (HU)	173.9 ± 21.3	151.5 ± 20.3	152.2 ± 22.4	*< 0.0001*	*< 0.0001*	0.27
Aorta (HU)	161.9 ± 23.2	143.8 ± 19.2	142.7 ± 22.6	*< 0.0001*	*< 0.0001*	0.51
Spleen (HU)	118.1 ± 11.9	107.3 ± 11.7	106.8 ± 16.0	*< 0.0001*	*< 0.0001*	0.62
Lower abdomen						
Subcutaneous fat (HU)	− 118.0 ± 8.7	− 109.4 ± 10.2	− 111.5 ± 10.4	*< 0.0001*	*< 0.0001*	0.51
Psoas muscle (HU)	59.3 ± 8.2	58.6 ± 5.4	59.5 ± 6.0	0.16	0.93	0.21

### Qualitative analysis

**Fig. 5:**
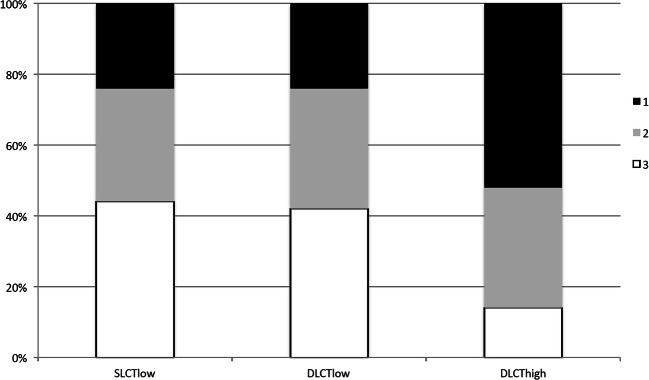
Results of the qualitative analysis showing the percentage of amount of received ratings, with best image quality observed for DLCT_high_. Image quality for DLCT_low_ was equal to SLCT_low_

No significant image quality differences were observed between SLCT_low_ and DLCT_low_ (*p* = 0.97). Overall, best image quality was observed for DLCT_high_ (mean rank ± SD, 1.62 ± 0.62) in comparison to SLCT_low_ (2.2 ± 0.71) and DLCT_low_ (2.18 ± 0.72) with *p* < 0.01 (Fig. 5).

ICC for quantitative inter-rater agreement was on average 0.89. Interobserver agreement for qualitative analysis was moderate with Cohen’s kappa of 0.42 according to the classification of Landis and Koch [14].

## Discussion

The necessity to increase the tube potential to at least 120 kVp for spectral imaging with current DLCT results in an increase of patient radiation exposure, compared with conventional single-layer acquisitions performed at 100 kVp. As a compensation tube current-time product might be adapted to achieve equivalent CTDI_vol_ to standard-, reduced-, or low-dose SLCT protocols. The results in this study show that patient radiation exposure can be restored to levels close to that of reduced-dose SLCT with comparable image quality by adjusting automated exposure control (i.e., DRI). Image quality was comparable between DLCT_low_ and SLCT_low_ despite the changes in tube potential and the resulting changes in tube current-time product. Thus, a notable dose reduction due to adjusted automatic exposure control can be achieved in DLCT protocols compared to standard protocols, but further reductions might be possible by fine-tuning the lower threshold for tube current-time product.

Previously, Van Ommen et al [15] had already shown that DLCT can be used for a broad range of applications without increasing patient radiation exposure compared to normal-dose SLCT. However, neither quantitative nor qualitative image quality was evaluated in this study. Another aspect of our study was the adaptation of DLCT to match previous low-dose SLCT_low_ with DLCT_low_, which shows lower radiation doses than the current literature [15, 16]. Moreover, the results show that even further dose reduction could be possible, especially in the thorax region where the automated exposure control reached the lower threshold of tube current-time product set in the acquisition protocol. According to the presented results, further dose reduction might be achieved by reducing the lower limit of the tube current from 65 to 55 mAs. According to Nagayama et al [17], DLCT showed a lower radiation dose than SLCT at equal tube potential of 120 kVp. However, with regard to image quality of conventional DLCT images, which is currently still the most frequently used reconstruction in clinical settings, no comparison was made with SLCT. Conventional CT images of dose equivalent DLCT and SLCT were already compared in a phantom study showing higher CNR but higher noise for DLCT compared to SLCT for 80–140 kVp, which was partly in agreement with the results presented here [18]. It should also be noted that despite matched CTDI_vol_ between SLCT_low_ and DLCT_low_, as configured in the acquisition protocol (Table 1), the resulting CTDI_vol_ for DLCT_low_ was higher than that for the SLCT_low_ (Table 2), which could be connected to the lower limit for the tube current mentioned above.

In general, the aspect of patient radiation exposure in spectral imaging has already been discussed in previous studies. No radiation dose increase is necessary for dual-source, dual-energy scans without compromises in image quality of the thorax and abdomen [19–21]. Rapid voltage switching dual-energy acquisition has shown ambiguous results. Singh et al showed dose equivalence to dual-source, dual-energy acquisition but with inferior image quality whereas other authors state that rapid voltage switching acquisition results in higher patient’s radiation [22, 23].

One limitation of the study is that the patients included in the quantitative evaluation were not scanned with all acquisition protocols, as 6 out of 31 patients were only scanned with two of the three protocols. As the quantitative evaluation did not rely on pairwise comparisons, but used an unbalanced design instead, it was possible to include patients where one of the acquisitions was missing as well as to include multiple acquisitions of the same patient. This was done to increase the statistical power of the results, as the overall number of patients included in this study was relatively low, which is another limitation. Because of the limited number of patients, the transferability of the obtained results to clinical practice may be limited as well. One of the reasons for the limited number of patients in this study is the restriction to a single acquisition protocol, which, even though it is the most frequently used acquisition protocol at that CT scanner, limits the overall number of eligible patients and also limits the transferability of the obtained results to other acquisition protocols. However, comparing different protocols at once would have led to an unnecessary complexity of the evaluation, as additional variables would have to be considered. This is also true for the inclusion of arterial-phase imaging, which is more susceptible to changes in patient habitus, cardiac output, and underlying disease (e.g., differences in enhancement patterns for different tumors, as evident in pancreatic carcinoma [24]). While the results achieved in this study are specific for the acquisition protocol and body region, and obtained from a limited number of patients, and reductions in patient radiation exposure may be larger or smaller for other protocols or patient collectives, which has to be the subject of further evaluation in the future, we do expect that the results are nonetheless fairly generalizable, as we chose to evaluate a single, widely applicable acquisition protocol that makes up the bulk of acquisitions at the evaluated CT scanner. Even though there is no clear consensus on the definition of low dose, with an average patient dose of 60% below the applicable DRL, the evaluated acquisition protocol in this study qualifies as “reduced dose” [25].

Another limitation of this study and the DLCT acquisition protocols with increased tube potential might be the inferior conspicuity of vessel structures at higher tube potential, as mean photon energies closer to the k-edge of iodine provide improved image contrast [26]. However, with the additional possibility of spectral post-processing provided by DLCT, vessel delineation might be improved with virtual monochromatic images at lower virtual photon energies [27]. Furthermore, only conventional images of DLCT were analyzed in this study, even though spectral imaging was the main reason for the tube potential increase. Nonetheless, the number of potential spectral post-processing applications is very large and the quantification of the potential benefit from the additional spectral information for clinical routine is beyond the scope of this study.

Regarding the results, the inter-reader agreement for qualitative analysis was found to be moderate. However, this might be a consequence of the forced choice method used for evaluation combined with the low number of available choice (3 images). In consequence, a disagreement of ranking for one patient always consists of at least two further mismatches, resulting in a moderate Cohen’s kappa. As all CT images were of diagnostic quality, the forced choice method was able to provide a side-by-side comparison, allowing to identify even small differences in image quality that might otherwise remain hidden with more conventional methods, like rating image quality on a Likert scale. Moreover, the quantitative analysis found differences in the delineation of vasculature as shown by the differences in CT numbers measured in the vessels. These differences did not lead to significant differences in subjective image quality, as delineation of vasculature is but one aspect of overall image quality. While Singh et al or Tabari et al [22, 28] suggest to link the evaluation of image quality to the clinical task of lesion detection, this approach was not applicable to this study as not all patients had lesions in all three evaluated body regions. In consequence, future studies may evaluate different aspects of image quality to provide a more complete evaluation for different clinical tasks.

## Conclusion

Overall, image quality of DLCT_low_ was comparable to SLCT_low_ and a low-dose acquisition was possible using dual-layer technique, which has the additional benefit of providing spectral information. With DLCT_low_, CTDI_vol_ could be reduced by 28% compared to DLCT_high_, but remained above that of SLCT_low_. Further reduction of patient radiation exposure for the thorax seems possible.

## Abbreviations

AP: Anterior-posterior
CNR: Contrast-to-noise ratio
CT: Computed tomography
CTDI_vol_: Volumetric computed tomography dose index
DLCT: Dual-layer spectral CT
DLP: Dose-length product
DRI: Dose right index
DRL: Diagnostic reference levels
ICC: Intraclass-correlation coefficient
ROI: Region of interest
SD: Standard deviation
SLCT: Single-layer CT
SSDE: Size-specific dose estimate
VMSI: Virtual monochromatic spectral imaging
